# Zebrafish (*Danio rerio*) behaviour is largely unaffected by elevated pCO_2_

**DOI:** 10.1093/conphys/cow065

**Published:** 2016-12-29

**Authors:** Laura E. Vossen, Fredrik Jutfelt, Arianna Cocco, Per-Ove Thörnqvist, Svante Winberg

**Affiliations:** 1 Uppsala University, Department of Neuroscience, Husårgatan 3, 75124 Uppsala, Sweden; 2 Norwegian University of Science and Technology, Department of Biology, Høgskoleringen 5, Realfagbygget, Trondheim, Norway

**Keywords:** Behavioural lateralization, detour, gabazine, ocean acidification, open field, zebrafish

## Abstract

Ocean acidification, the decrease in ocean pH caused by anthropogenic emission of carbon dioxide, can cause behavioural disturbances in marine teleost species. We investigated whether AB-strain zebrafish (*Danio rerio*) show similar behavioural disturbances in the presence of elevated CO_2_, because this model species could open up a toolbox to investigate the physiological and neurological mechanisms of CO_2_ exposure. We found no effect of elevated CO_2_ (~1600 μatm) on the behaviour of zebrafish in the open field test, indicating that zebrafish are largely insensitive to this elevated CO_2_ level. In the detour test of lateralization, however, zebrafish exposed to elevated CO_2_ swam more often to the right, whereas individuals exposed to control CO_2_ (~400 μatm) had no preference for left or right. This may indicate that some behaviours of some freshwater fishes can be altered by elevated CO_2_ levels. Given that elevated CO_2_ levels often occur in recirculating aquaculture and aquarium systems, we recommend that dissolved CO_2_ levels are measured and, if necessary, the aquarium water should be aerated, in order to exclude CO_2_ level as a confounding factor in experiments.

## Abstract (precis)

End-of-century CO_2_ levels can disturb the behaviour of marine fish. We exposed freshwater zebrafish to elevated CO_2_, which affected turning preferences but not activity and anxiety levels. This indicates that some behaviours of freshwater fishes can be altered by elevated CO_2_ in a similar manner to marine fishes.

## Introduction

Anthropogenic emissions of carbon dioxide are acidifying the world's water bodies. It has been estimated that ocean pH has already dropped 0.1 pH unit since the onset of the industrial revolution, and a further decrease in ocean pH of 0.2–0.3 pH units is expected by the end of the century (Haugan and Drange, 1996; Rhein *et al*., 2013). Recent experiments on marine teleost fish reveal alarming behavioural impairments upon exposure to near-future CO_2_ levels, including the impairment of olfactory, visual and auditory responses, increased activity, increased boldness, reduced learning and changes in behavioural lateralization (reviewed by Munday *et al*., 2009; Briffa *et al*., 2012; Clements and Hunt, 2015). Also, ‘CO_2_-resilient’ species have been reported. In Atlantic cod (*Gadus morhua*), larval swimming kinematics are unaffected and adults strongly avoid CO_2_ and predator odour despite long-term CO_2_ exposure (Maneja *et al*., 2012; Jutfelt and Hedgärde, 2013). The hypothesized mechanism causing these behavioural disturbances is that the major inhibitory neurotransmitter, γ-aminobutyric acid (GABA), reverses its function in the presence of an elevated partial pressure of carbon dioxide (pCO_2_; Nilsson *et al*., 2012). To avoid acidosis, fish take up bicarbonate ions (HCO_3_^−^) into the gill cells and extrude chloride ions (Cl^−^) into the water (Brauner and Baker, 2009). This alters the plasma ion concentrations, which in turn can affect the electrochemical gradients over the cell membranes. Therefore, when GABA binds to the GABA_A_ receptor, the reduced extracellular Cl^−^ concentrations may reduce the electrochemical gradient for Cl^−^ influx, while the increased intracellular HCO_3_^−^ concentrations may lead to efflux of these anions and cause depolarization rather than the normal hyperpolarization. In accordance with the ‘GABA hypothesis’, treatment with the GABA_A_ receptor antagonist gabazine restored the behaviour of CO_2_-exposed fish in the majority of studies (Nilsson *et al*., 2012; Chivers *et al*., 2013; Chung *et al*., 2014; Lai *et al*., 2015).

Whether freshwater fish are affected by elevated pCO_2_ via the same mechanism has not yet been sufficiently investigated (Leduc *et al*., 2013; Ou *et al*., 2015). Freshwater pH and pCO_2_ vary more over space and time (e.g. because of heavy rainfall or snow melting, biological respiration, lower buffering capacity) and therefore it has been argued that freshwater fish might have evolved greater tolerance to pH fluctuations (Ishimatsu *et al*., 2005; Leduc *et al*., 2013; Heuer and Grosell, 2014). In addition, freshwater fish have very robust NaCl uptake mechanisms for ionoregulation, which are linked to H^+^ and HCO_3_^−^ secretion. However, even in freshwater fish hypercapnic acidosis may be linked to uptake of water HCO_3_^−^ in exchange for Cl^−^, because in freshwater-raised salmon smolts elevated CO_2_ results in lower plasma Cl^−^ concentrations (Fivelstad *et al*., 2003a). Thus, elevated pCO_2_ may potentially impair GABA functioning through the same mechanism as in marine fish. Ultimately, rising freshwater pCO_2_ could potentially threaten freshwater fish populations in the same way as in marine ecosystems (Munday *et al*., 2010); however, much less is known about future changes in freshwater pCO_2_ compared with predictions for ocean pCO_2_ (Hasler *et al*., 2016).

To investigate whether elevated CO_2_ can also affect the GABA system of a freshwater fish species, we exposed adult zebrafish (*Danio rerio*) to control (~400 μatm) or elevated CO_2_ (~1600 μatm). We chose zebrafish for the following three reasons. Firstly, it is a widely used model organism in genetics and neuroscience, and this species would therefore open up a toolbox to investigate the neural, cellular and molecular mechanisms altered by CO_2_ (Briggs, 2002; Norton and Bally-Cuif, 2010). Secondly, the natural habitat of the zebrafish consists of slow-moving pools and rice paddies, with reported pH as low as 5.9 and as high as 8.1 (Engeszer *et al*., 2007), environments where pCO_2_ can reach high levels. If this species familiar with high CO_2_ concentrations is affected, then this will probably also be the case for many more freshwater fish. Thirdly, laboratory strains of zebrafish are usually housed in recirculating rack systems, in which CO_2_ concentrations can quickly rise because of respiration of the fish or microbial activity in tanks and filters. We have included a set of CO_2_ measurements taken in seven independent recirculating aquatic housing systems holding zebrafish, one of which measured 1200 μatm (Table 1). The CO_2_ level might therefore constitute an unexplained factor in laboratory experiments with zebrafish. In larger recirculating tank systems, such as those used in aquaculture, dissolved CO_2_ can even reach levels up to 10 000 μatm (Fivelstad *et al*., 2003b).

**Table 1:** Survey of fish density, pCO_2_, pH, temperature and conductivity in seven independent zebrafish aquatic housing systems at four different anonymous biomedical facilities in Sweden

Aquatic housing system	Manufacturer	Fish density in tank	pCO_2_ (μatm)	pH	Temperature (°C)	Conductivity (μS cm^−1^)
1	Aquaneering	12 in 9 litres	470	8.84	27.0	422
2	Tecniplast	30 in 9 litres	450	7.41	27.8	495
3	Aquaneering	30 in 9 litres	720	7.20	28.0	600
4	Aqua medic T 2001 HC	20 in 3 litres	830	7.20	28.0	874
5	Pentair aquatic ecosystems	61 in 10 litres	720	7.8	28.2	n.a.
6	Aquatic habitats (duo system)	5 in 3 litres	730	8.0	27.8	n.a.
7	Aquatic habitats (standalone)	149 in 10 litres	1200	7.5	26.0	n.a.
7	Aquatic habitats (standalone)	3 in 3 litres	820	7.5	26.0	n.a.

Abbreviations: n.a., not assessed; and pCO_2_, partial pressure of carbon dioxide.

The pCO_2_ level used in this study (1600 μatm) was chosen for being higher than what zebrafish are likely to experience in the laboratory or in the field (Engeszer *et al*., 2007), while not exceeding the maximum of 2000 μatm predicted by current models for long-term ocean pCO_2_ (Zickfeld *et al*., 2013). The open field test used in this study is a widely used test to investigate exploratory behaviour and anxiety as well as activity level (Blaser and Gerlai, 2006; Grossman *et al*., 2010; Maximino *et al*., 2010; Stewart *et al*., 2010), behaviours which have been reported to be affected in the presence of elevated CO_2_ levels (Munday *et al*., 2010, 2013; Cripps *et al*., 2011). The detour test was chosen because it provides a test of brain function for different decision-making tasks (Vallortigara and Rogers, 2005). Disruptions in behavioural lateralization have already been shown for the coral reef fishes *Neopomacentrus azysron* (Domenici *et al*., 2012) and *Pomacentrus wardi* (Domenici *et al*., 2014), as well as for the temperate species the three-spined stickleback (*Gasterosteus aculeatus*; Jutfelt *et al*., 2013; Lai *et al*., 2015). Behavioural lateralization was unaffected by CO_2_ exposure in temperate wrasse (*Ctenolabrus rupestris*; Sundin and Jutfelt, 2015).

The present study investigated whether zebrafish of the AB strain show abnormal behaviour in open field and detour tests when exposed to elevated pCO_2_. Furthermore, we explored whether any behavioural disturbances could be reversed by treatment with the specific GABA_A_ receptor antagonist, gabazine.

## Materials and methods

### Experimental animals

Rearing, handling and experimental procedures were approved by the ethical committee on animal experiments of Uppsala, Sweden (ethical permit: C 55/13 to S.W.). All fish used in this experiment were adult wild-type (AB) zebrafish. They were ordered as eggs from ZIRC (University of Oregon, USA), bred at the Evolutionary Biology Center (Uppsala University, Uppsala, Sweden) and held at the Department of Neuroscience (Uppsala University) for at least 6 months before the start of the experiment. Fish were kept at 28**°**C with a 14 h–10 h light–dark photoperiod. Water quality was monitored for alkalinity (mean ± SD, 2.03 ± 0.09 mequiv l^−1^), total hardness (80 ppm), conductivity (mean ± SD, 426.01 ± 2.73 μS cm^−1^), nitrite (<1 mg l^−1^), nitrate (<10 mg l^−1^) and ammonium (mean ± SD, 0.15 ± 0.13 mg l^−1^). Fish were fed *ad libitum* once a day in the morning with flakes (Serasan flakes for tropical fish) and live *Artemia* nauplii.

### Experimental treatments

The experiment took place from February to April 2014. At the start of the experiment, a total of 120 adult fish were equally distributed over four 25 litre aquaria; 15 males and 15 females per tank. Two aquaria were connected to a header tank that was bubbled with air (control exposure), while the other two aquaria received water from a header tank that was bubbled with 100% CO_2_ gas using a solenoid valve controlled by a pH stat computer such that pCO_2_ was maintained at a target value of 1600 (Aqua Medic, Bissendorf, Germany). We realize that our experimental set-up would have benefited from the use of several header tanks per CO_2_ treatment (Riebesell *et al*., 2010; Moran, 2014). In all four aquaria, pCO_2_ was measured daily using an infrared dissolved CO_2_ meter (Qubit, Kingston, ON, Canada). The pCO_2_ of the control aquaria was 420.5 ± 57.3 μatm (mean ± SD) and the pCO_2_ of the elevated-CO_2_ aquaria was 1610.7 ± 277.8 μatm (mean ± SD). The pH was 8.31 ± 0.04 (mean ± SD; control pCO_2_) and 7.58 ± 0.09 (mean ± SD; elevated pCO_2_). The fish were kept in these exposure aquaria for 26–48 days to allow for potential acclimation. This is a longer duration than the exposure used for fish in many other studies, and larval clownfish (*Amphiprion percula*) show impairments already after CO_2_ exposure for 1 day (Munday *et al*., 2010).

On the day of behavioural testing, zebrafish from each exposure tank were randomly assigned to either an individual immersion treatment with the specific GABA_A_ receptor antagonist gabazine (4 mg l^−1^ in 4 litres of tank water for 30 min at 27°C) or control treatment (50 ml tap water in 4 litres of tank water for 30 min at 27°C). Immersion treatment tanks were prepared fresh on the morning of every testing day from cooled (4°C) stock solutions of gabazine and tap water (the ‘control stock’, cooled and thawed in the same way as the gabazine stock solution). Each immersion treatment tank was used by two or three individual fish during the testing day. After the behavioural tests (see next subsection), animals were euthanized in a benzocaine solution (500 mg l^−1^ buffered to pH 7.5) with ice, the spine was cut at the neck, and the gonads were dissected to confirm the sex of the individual.

### Behavioural tests

Fish were first tested in the open field test (118 individuals, 30 min) and subsequently in the detour test (68 individuals). Testing aquaria contained water of control pCO_2_, and tests were carried out within 3 h after catching from the home tank, long before fish recover from elevated pCO_2_ (Munday *et al*., 2010). The open field arenas were rectangular plastic tanks (30 cm width × 35 cm length) with white opaque walls, filled with 4 litres of water, resulting in 4 cm of water depth. An infrared light board (Noldus, Wageningen, The Netherlands) was placed under the arenas, and an overhead infrared camera (JVC SuperLoLux, Yokohoma, Japan) attached to a computer recorded fish activity. The testing room was sound and light proof and had ambient symmetrical lighting. Films were analysed with the automatic tracking software EthoVision XT10 (Noldus) at 25 frames s^−1^, collecting the following activity data: swimming speed; total distance moved; movement (duration moving/not moving); and mobility state (duration mobile, highly mobile and immobile; Grieco *et al*., 2010). The swimming speeds were recalculated into relative speeds by dividing the speed (in centimetres per second) by the individual's total length measurement. In order to quantify thigmotaxis (‘wall-hugging’), the arena was divided into a wall zone (defined as the outermost 4 cm of the arena) and an inner zone (the rest, or centre of the arena; Gerlai *et al*., 2000; Maximino *et al*., 2010). To quantify the number of home bases, the function ‘Heatmap Visualization’ inside Ethovision XT10 was applied to each individual trial, and the number of home bases were counted as the number of red and/or dark red areas in the arena, which are indicative of a long presence of the subject's centre point in that area (Stewart *et al*., 2010).

**Figure 1:**
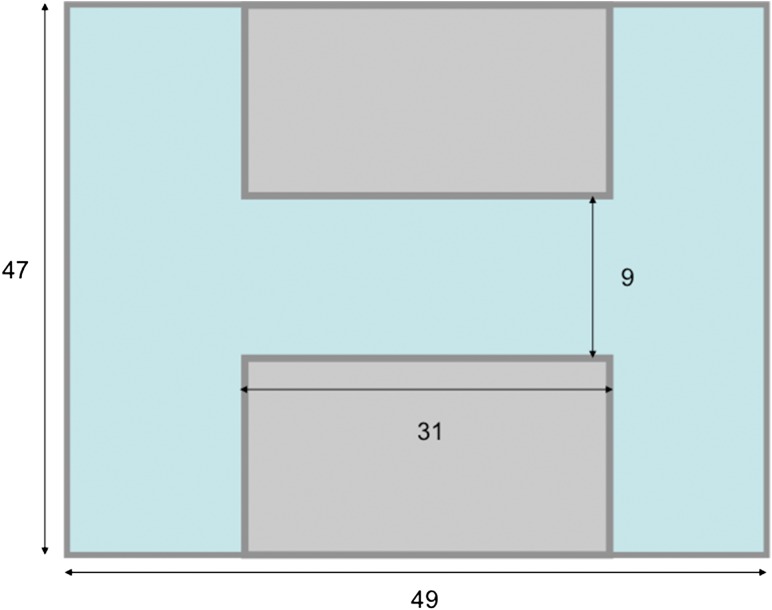
Schematic representation of the double T-chamber used for the detour tests of lateralization. Measurements are in centimetres. The figure is not drawn to scale.

In the detour test, a double T-chamber was used to evaluate the effect of elevated CO_2_ on behavioural lateralization (see Fig. 1; after Jutfelt *et al*., 2013). Most zebrafish readily swim through a double T-chamber from side to side, i.e. without any encouragement from researchers. If an individual had not made the first five choices within 5 min, the trial was discarded (an equal number of trials was discarded from control and elevated pCO_2_; binomial test for equality of proportions, χ^2^ = 0.605, *P* = 0.437). Individuals were introduced into the chamber in the middle of one long arm, and the first left or right turn was recorded as soon as they went more than halfway through the runway and then chose a left or right arm. Each individual's turning preference was measured 20 times post testing from video recordings. For easier comparison with previous studies, we calculated the relative lateralization index (*L*_R_), which reflects the preference to turn either left or right, for each fish as follows: [(number of right turns − number of left turns)/(number of right turns + number of left turns)] × 100. The absolute lateralization index (*L*_A_), which reflects the strength of any possible side bias regardless of this bias being to the left or the right, was calculated as the absolute value of the relative lateralization index (Bisazza *et al*., 1998).

### Measurements of pCO_2_ in zebrafish recirculating tank systems

The pCO_2_ was measured using an infrared dissolved CO_2_ meter (Qubit Biology Inc., Canada) inside holding tanks in seven independent zebrafish housing systems in four different biomedical laboratories (Table 1). Temperature, pH and conductivity were read off from housing system displays. For one housing system (number 7), two pCO_2_ measurements were performed in two different holding tanks, one with low and one with high fish density.

### Statistical analyses

Statistical analyses were performed using R language and environment for statistical computing and graphics, version 3.2.3 (R Development Core Team, 2013) and the R package ‘lme4’ (Bates *et al*., 2015; see also the Supplementary material).

## Results

### Open field test

**Figure 2:**
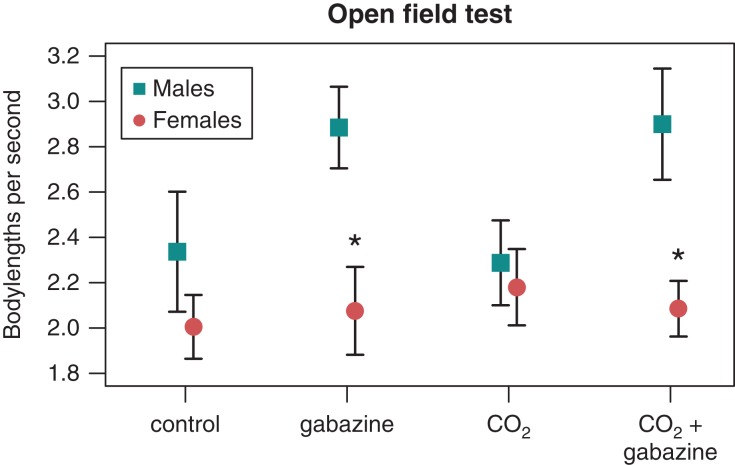
Relative swimming speed (mean ± SEM; in body lengths per second) in the open field test during the 30 min trial of male (blue squares) and female (red circles) zebrafish exposed to control pCO_2_ (~400 μatm; ‘control’), control pCO_2_ and gabazine (‘gabazine’), elevated pCO_2_ (~1600 μatm; ‘CO_2_’) and elevated pCO_2_ and gabazine (‘CO_2_ + gabazine’) for an average of 37 days. Asterisks indicate the significant increase in male swimming speed in response to gabazine treatment (*P* < 0.001).

Carbon dioxide exposure did not affect any of the activity variables [generalized linear mixed effects models (GLMMs), *P* > 0.05; Table S1]. There was a significant effect of sex [linear mixed effects model (LMM), *F*_1,113_ = 17.686, *P* < 0.001] and gabazine treatment (LMM, *F*_1,105_ = 5.763, *P* = 0.018), as well as a sex-by-treatment interaction (LMM, *F*_1,108_ = 5.561, *P* = 0.020) on the swimming speed (in fish lengths per second; Fig. 2 and Table S3) and distance moved in the open field test. Males swam faster than females in the gabazine treatments (gabazine/control pCO_2_ and gabazine/elevated pCO_2_; *post hoc* *t*-test, *t* = −4.6, *P* < 0.001) than in the drug control treatments (no gabazine/control pCO_2_ and no gabazine/elevated pCO_2_; *post hoc* *t*-test, *t* = −1.38, *P* = 0.169). Males were immobile for a shorter duration of time in the gabazine treatments (*post hoc* *t*-test, *t* = 3.44, *P* < 0.001). The durations of time moving, not moving, mobile and highly mobile were not affected by any of the explanatory variables (GLMMs, *P* > 0.05). Gabazine treatment increased thigmotaxis (GLMM with binomial errors, Wald χ^2^ = 5.288, *P* = 0.021; Table S4), whereas sex and CO_2_ exposure level had no effect on thigmotaxis (*P* > 0.2). The number of home bases was not influenced by CO_2_ exposure, drug treatment or sex (generalized linear model with Poisson error distribution, *P* > 0.05).

### Detour test

**Figure 3:**
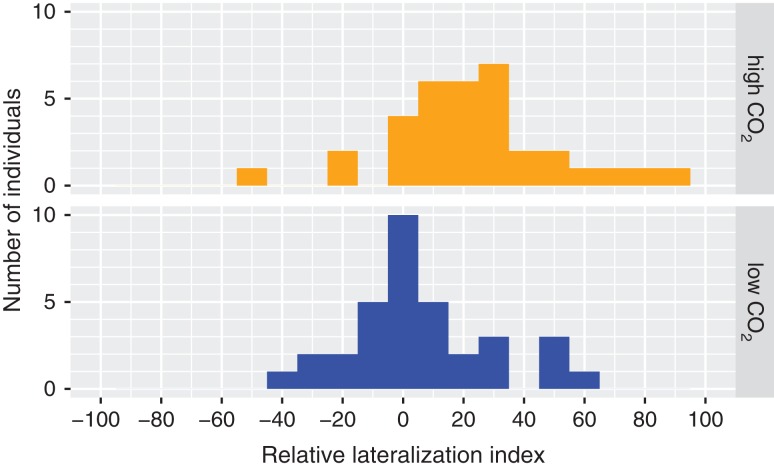
The relative lateralization index (*L*_R_, a measure that reflects the preference to turn either left or right) of zebrafish exposed to control pCO_2_ (~400 μatm) and elevated pCO_2_ (~1600 μatm). Frequency distributions of the number of fish with each *L*_R_ are shown. An *L*_R_ of −100 indicates all left turns and no right turns, an *L*_R_ of 0 corresponds to an equal number of left and right turns, and an *L*_R_ of 100 indicates that all turns were to the right. Carbon dioxide exposure significantly increased the proportion of right turns.

**Figure 4:**
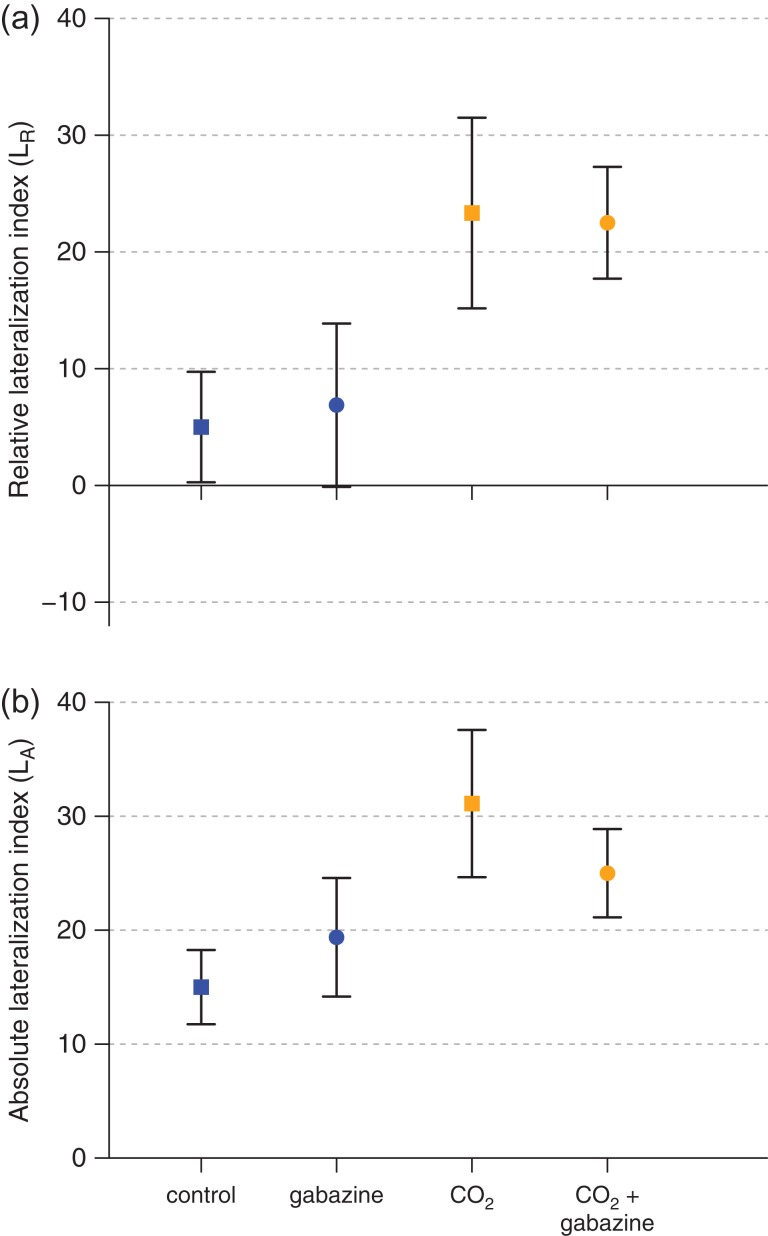
Mean (±SEM) relative (**a**) and absolute (**b**) lateralization index in the detour test for adult zebrafish exposed to control pCO_2_ (~400 μatm; ‘control’, blue squares), control pCO_2_ and gabazine (‘gabazine’, blue circles), elevated pCO_2_ (~1600 μatm; ‘CO_2_’, orange squares) and elevated pCO_2_ and gabazine (‘CO_2_ + gabazine’, orange circles) for an average of 37 days. The relative lateralization index (*L*_R_) reflects the preference to turn either left (negative *L*_R_) or right (positive *L*_R_), whereas a higher absolute lateralization index indicates a stronger side preference (irrespective of the left or the right side). Carbon dioxide exposure significantly increased the proportion of right turns.

Carbon dioxide exposure significantly increased the proportion of right turns in the detour test (GLMM with binomial errors, Wald χ^2^ = 8.1697, *P* = 0.0043; Tables S2 and S5), whereas gabazine treatment and sex did not influence the proportion of right turns. The relative lateralization index (*L*_R_) was 5.44 ± 4.08 in control CO_2_ and 22.94 ± 4.81 in elevated CO_2_ (mean ± SEM; Fig. 3). The absolute lateralization index (*L*_A_) was 16.91 ± 2.99 in control CO_2_ and 28.24 ± 3.86 in elevated CO_2_ (mean ± SEM; Fig. 4).

## Discussion

Exposure to elevated levels of carbon dioxide (1611 μatm) did not alter the behaviour of zebrafish in the open field test, which may indicate that adult zebrafish are mostly insensitive to this pCO_2_. Nonetheless, zebrafish behaviour might be altered by even higher levels of CO_2_ or in other life stages, warranting further investigation. Gabazine treatment affected male and female behaviour differently in the open field test. Males became significantly more active after gabazine treatment, whereas no effect of gabazine on the activity of female zebrafish was detected. Sex differences in the dynamic pattern of hyperactivity have also been observed in response to the partial inverse GABA_A_ receptor agonist FG-7142 (López-Patiño *et al*., 2008). Ou *et al*. (2015) studied the effects of CO_2_-induced acidification in freshwater-reared pink salmon and found that fish reared at 2000 μatm CO_2_ displayed less thigmotaxis than control fish and that gabazine treatment increased thigmotaxis. The present study verified the positive effect of gabazine on thigmotaxis; however, we did not detect an effect of 1611 μatm pCO_2_ on thigmotaxis despite reasonable sample size, which suggests that this behaviour is robust to this particular pCO_2_ in zebrafish.

Elevated pCO_2_ did have an effect on the behaviour of both male and female zebrafish in the detour test. Zebrafish exposed to elevated pCO_2_ turned more often to the right compared with zebrafish from control pCO_2_, which did not have a turning bias. Zebrafish possess a pattern of lateralization typical for tetrapods, where right frontal retinal fixation is associated with a period in which response (in particular escape) has to be inhibited until a decision has been taken about the nature of what is being viewed, such as when searching for a hidden predator (Miklósi *et al*., 1997; Facchin *et al*., 1999; Miklósi and Andrew, 2006). The left eye system is used to assess whether an object is novel or not in low-risk situations (Miklósi and Andrew, 2006). In our experimental chamber, the CO_2_-exposed zebrafish that swam more often to the right might have been driven more by stimuli from their right frontal field, because there was no stimulus in front of the runway. After they inspected the right side with the right eye and established that it did not contain a threat, they swam into the right arm. However, the fish from control CO_2_ showed no side preference and were therefore probably using both eyes to an equal extent. The greater use of the right eye system could therefore mean that CO_2_-exposed fish were in a higher state of alertness than control zebrafish.

Elevated pCO_2_ can be an indicator of poor water quality, which is stressful to fish (Huntingford *et al*., 2006). Zebrafish can sense small changes in pCO_2_ in the environment via neuroepithelial cells located in the gills (Qin *et al*., 2010), and adult zebrafish also increase their ventilation amplitude in response to a pCO_2_ of 1 mmHg (~1300 μatm; Vulesevic, 2006). In future experiments, it would be interesting to measure cortisol concentrations in response to CO_2_ exposure. The response cannot be explained by altered GABA function from the chronic effects of CO_2_, as suggested in marine fishes (Nilsson *et al*., 2012), because gabazine treatment did not affect lateralization, and the elevated CO_2_/gabazine group did not show restored lateralization behaviour.

Disturbances in behavioural lateralization might hamper an individual's schooling ability, which is important for survival in the wild (Bisazza and Dadda, 2005). Several studies have investigated behavioural lateralization in the context of ocean acidification, with somewhat differing results, as discussed by Sundin and Jutfelt (2015).Our results perhaps best resemble those of Welch *et al*. (2014), who also reported an increase in the number of right turns. However, their juvenile damselfish shifted from a left bias in control pCO_2_ to equal numbers of left- and right-biased fish in elevated pCO_2_, rather than from no bias to a right bias in the presence of elevated pCO_2_ (our study).

In recirculating tank systems, CO_2_ levels can quickly rise as a result of respiration by fish and micro-organisms and the high solubility of this gas in water. We performed pCO_2_ measurements in seven independent zebrafish housing systems (Table 1) and found that five had a pCO_2_ >700 μatm. The tank with the highest pCO_2_ level (1200 μatm) also had the highest density of fish. We therefore advise researchers to measure dissolved CO_2_ levels in their tank systems and to aerate tanks when the relative biomass is high. Some species might be more tolerant to CO_2_ than others; therefore, more research is needed to establish the exact upper limit of pCO_2_ in the zebrafish.

Owing to a behavioural shift in lateralization in high pCO_2_, zebrafish could be considered as a model species in the context of aquatic acidification research. We do not yet know whether the mechanisms behind the behavioural effects seen here are similar to the mechanisms in marine fishes. Other behavioural tests could be performed on zebrafish in elevated pCO_2_ to verify the results of the detour test. In addition, a better understanding is needed of the mechanisms regulating freshwater pCO_2_ and how freshwater pCO_2_ responds to climate change (Hasler *et al*., 2016).
